# Using the CHOPS score to evaluate the severe cerebral venous sinus thrombosis progression risk after anticoagulation

**DOI:** 10.3389/fneur.2026.1848582

**Published:** 2026-08-26

**Authors:** Ya-Qi Liu, Wei-Dong Xu, Guan-Hua Zhang, Xiao-Fei Huang, Yun Xu, Qiu-Jing Wang

**Affiliations:** 1 Department of Cerebrovascular Surgery, The Third Affiliated Hospital of Sun Yat-sen University, Guangzhou, China; 2 Tongji Junshan Neuroscience Center, Tongji Hospital, Tongji Medical College, Huazhong University of Science and Technology, Wuhan, Hubei, China

**Keywords:** anticoagulation, progression, risk factor, score, severe cerebral venous sinus thrombosis

## Abstract

**Objective:**

This aimed to evaluate the risk of severe cerebral venous sinus thrombosis (SCVST) progression after anticoagulation using clinical and radiological data.

**Methods:**

Clinical and radiological data from 199 SCVST patients were collected and analyzed in this study. Univariate and multivariate logistic regression analyses were conducted for several factors, including age, gender, pregnancy and puerperium, oral contraceptive use, coma state, hemiplegia, sensory dysfunction, cerebral infarction, cerebral hemorrhage, and sinus involvement. Subsequently, an evaluation system was developed based on the odds ratio (OR), and the receiver operating characteristic (ROC) curve was used to determine the prediction accuracy of this evaluation system in another cohort of 32 SCVST patients.

**Results:**

Pregnancy and puerperium (OR = 5.525, *p* = 0.001), oral contraceptive use (OR = 28.720, *p* = 0.003), coma at admission (OR = 3.890, *p* < 0.001), cerebral hemorrhage (OR = 2.151, *p* = 0.038), and number of occluded sinuses (OR = 1.708, *p* < 0.001) were found to be independent risk factors predicting the SCVST progression. The CHOPS score comprising the five independent factors (coma at admission (C), cerebral hemorrhage (H), oral contraceptive use (O), pregnancy and puerperium (P), and the number of occluded sinuses (S)) was constructed, which achieved an area under the curve of 0.873.

**Conclusion:**

Pregnancy and puerperium, oral contraceptive use, coma at admission, cerebral hemorrhage, and the number of occluded sinuses are independent risk factors predicting the SCVST progression after anticoagulation. The developed CHOPS score can accurately predict SCVST progression; thus, endovascular or surgical treatment may be considered after anticoagulation when clinically necessary.

## 1 Introduction

### 1.1 Background

Cerebral venous sinus thrombosis (CVST) is a relatively rare cerebrovascular disease, with an incidence estimated at 1.32 per 100,000 person-years (1). Clinical practice, the symptoms and outcomes of CVST patients are highly heterogeneous. Mild CVST cases only present with mild headache or no symptoms. However, severe CVST (SCVST) may potentially cause infarction, hemorrhage, and cerebral herniation, which lead to poor patient outcomes (2). Currently, the mortality of SCVST has been reported to be in the range of 5%–30% (3).

Although endovascular and surgical treatments, including mechanical thrombectomy, thromboaspiration, and decompressive craniotomy, have been considered to be effective in treating SCVST, systemic anticoagulation is still the first-line treatment (4). However, data indicate that some patients receiving anticoagulation treatments still progress rapidly and thus require endovascular intervention, especially those with large and extensive SCVST (5–9). This underscores the need to identify patients who are likely to be progressive after anticoagulation and receive endovascular and surgical treatments sooner to prevent poor prognosis.

This study aimed to identify the predictors of SCVST progression, and thus endovascular or surgical treatment may be considered when clinically necessary.

## 2 Methods

This was a retrospective study, and it was approved by the ethics committee of The Third Affiliated Hospital of Sun Yat-sen University. We conducted this study following the guidelines and regulations of the ethics committee.

### 2.1 Patients

This was a retrospective study conducted from 1 January 2016 to 30 June 2022. Informed consent was obtained from each patient or their relatives. In total, clinical data from 199 SCVST patients were retrospectively collected at The Third Affiliated Hospital of Sun Yat-sen University. The inclusion criteria were cerebral hemorrhage after CVST, mental status impairment, coma (Glasgow Coma Scale (GCS) < 9), thrombosis in the deep cerebral venous system or cortical venous thrombosis, and intracranial hypertension. Of note, CVST patients meeting one or more of the above criteria were defined as SCVST and included in this study. Exclusion criteria included not agreeing to have MRV, CTV, or digital subtraction angiography (DSA) examination, having received endovascular and surgical treatment of CVST before admission, those with cerebral herniation before admission, and those with contraindications to anticoagulation. The definitions of SCVST and the inclusion criteria adopted in our study are similar to those utilized by Yang et al. (10)

To validate the results, clinical data from 32 SCVST patients were collected from July 2022 to June 2024 and served as the validation cohort.

### 2.2 Anticoagulation therapy

The SCVST patients enrolled in this study received anticoagulation following admission, and the anticoagulation procedure was as follows. In the first week, the patients were administered dalteparin 100 unit/kg subcutaneously twice daily. They were then prescribed warfarin to maintain INR between 2 and 3 for at lease 6 months. The anticoagulation procedure adopted in this study was similar to that reported by Misra et al. (11)

### 2.3 Clinical and radiological evaluation

Clinical data including gender, age, pregnancy history, and oral contraceptive use were recorded at admission. In addition, the conscious state and modified Rankin Scale (mRS) were evaluated during the treatment and follow-up periods. The patients’ CT, MRI, and DSA examinations before and after treatment were analyzed. The patients’ radiological data obtained before and after treatment were recorded and analyzed, including the sites of thrombosis, cerebral infarction and cerebral hemorrhage.

### 2.4 Endovascular and surgical treatments

The enrolled patients were initially administered anticoagulation after admission. For patients with progressive disturbance of consciousness (GCS score decrease >3 points), neurological impairment (NIHSS score increase >5 points) or cerebral hemorrhage increase >10 mL in reexamination CT scan, patients were included in the progressive group, and endovascular or surgical treatment may be considered when clinically necessary. Patients without progression during the 2 month follow-up, they were classified as stable. For patients with progressive disturbance of consciousness or worsening neurological deficits, follow-up CT scans were performed immediately. For patients without progression, follow-up CT scans were performed twice weekly during the first month and once weekly during the second month.

### 2.5 Statistical analysis procedure

All statistical analyses were performed using SPSS 22.0 (IBM Inc., Chicago, IL). Continuous variables in the progressive and stable groups were expressed as the mean ± SD, whereas categorical variables were presented as counts with percentages. The factors (i.e., age, gender, previous history, physical signs at admission, and radiological data) influencing the efficacy of anticoagulation were analyzed using logistic regression. Those with a significant association (*p* < 0.1) with progressive SCVST in the univariate analysis were included in the multivariate regression analysis. The multivariate regression results with a *p*-value <0.05 were considered to be significantly associated with progressive SCVST. A score was developed to determine the progressive SCVST risk using variables identified to be significant in the multivariate regression analysis, with the OR values of variables used to allocate scores.

To validate the performance of the developed scoring system, the receiver operating characteristic (ROC) and area under the curve (AUC) were calculated to evaluate its sensitivity and specificity, using data from a cohort of 32 SCVST patients enrolled between July 2022 and June 2024. The statistical analysis procedures were adopted from a previous study (12).

## 3 Result

### 3.1 Baseline characteristics of patients

A total of 199 patients were enrolled, with a mean age of 37.4 years. A total of 110 patients (55.3%) were male. A total of 27 (13.6%) were pregnant and within the puerperium period, and 10 (5.0%) had used oral contraceptives within 30 days before admission. Before anticoagulation, 17 (8.5%) SCVST patients had deep cerebral venous system or cortical venous thrombosis, 105 (52.8%) had cerebral hemorrhage, 81 (40.7%) had altered consciousness, and 172 (86.4%) had intracranial hypertension. Notably, 89 patients (44.7%) achieved progressive SCVST after anticoagulation, and thus received endovascular or surgical treatment.

Within the 89 progressive patients, 37 patients received endovascular treatment and 15 (40.5%) patients had good outcomes (mRS ≤ 2) after 6-month follow-up. 45 patients received endovascular treatment and 14 (31.1%) patients had good outcomes. 7 patients received endovascular and surgical treatments successively, and 1 patient (14.3%) had a good outcome.

### 3.2 Statistical analysis

The univariate logistic regression results for each variable are shown in Table 1. On average, the mean age of patients in the progressive group was higher than that of patients in the stable group, but the difference was not significant (38.61 years vs. 36.41 years, *p* = 0.304). There were more males in the stable group than in the progressive group (59.09% vs. 50.56%), but the difference was not significant (*p* = 0.230). Further analysis revealed that the proportions of pregnancy and puerperium, and oral contraceptive use were significantly lower in the stable group compared with the progressive group (7.27% vs. 21.35 and 0.91% vs. 10.11%, *p* = 0.006 and *p* = 0.019, retrospectively).

**Table 1:** Logistic regression of clinical and radiological data from 199 SCVST patients.

Variable	Stable group	Progressive group	Univariate		Multivariate			
			*p*-value	OR	*p*-value	OR	*B*	95% CI
Age (year)	36.41 ± 16.28	38.61 ± 13.23	0.304	1.010				
Gender (%)			0.230	0.708				
Male	65 (59.09)	45 (50.56)						
Female	45 (40.91)	44 (49.44)						
Pregnancy and puerperium (%)	8 (7.27)	19 (21.35)	0.006	3.461	0.001	5.525	1.709	1.987–15.363
Oral contraceptive use (%)	1 (0.91)	9 (10.11)	0.019	12.262	0.003	28.720	3.358	3.204–257.435
Coma (%)	29 (26.36)	52 (58.43)	<0.001	3.925	<0.001	3.890	1.358	1.925–7.860
Hemiplegia (%)	36 (32.73)	49 (55.96)	0.002	2.518				
Sensory dysfunction (%)	4 (3.64)	10 (11.24)	0.047	3.354				
Cerebral infarction (%)	47 (42.73)	42 (47.19)	0.529	1.198				
Cerebral hemorrhage (%)	50 (45.45)	55 (61.80)	0.022	1.941	0.038	2.151	0.766	1.044–4.431
Superior sagittal sinus involved (%)	55 (50.00)	67 (75.28)	<0.001	3.045				
Inferior sagittal sinus involved (%)	8 (7.27)	8 (8.99)	0.659	1.259				
Torcular herophili involved (%)	8 (7.27)	16 (17.98)	0.025	2.795				
Deep vein involved (%)	5 (4.55)	22 (24.72)	<0.001	6.896				
Tentorial sinus involved (%)	16 (14.55)	31 (34.83)	0.001	3.140				
Transverse sinus involved (%)								
Unilateral	70 (63.64)	49 (55.06)	0.713	0.888				
Bilateral	7 (6.36)	14 (15.73)	0.080	2.538				
Sigmoid sinus involved (%)								
Unilateral	51 (46.36)	45 (50.56)	0.313	1.348				
Bilateral	4 (3.64)	8 (8.99)	0.085	3.056				
Cortical vein involved (%)	5 (4.55)	12 (13.48)	0.138	1.981				
Number of sinuses involved	2.14 ± 1.27	3.17 ± 1.66	<0.001	1.618	<0.001	1.708	0.535	1.341–2.176

At admission, the stable group had a significantly lower proportion in the coma state than the progressive group (26.36% vs. 58.43%, *p* < 0.001). Moreover, the proportions of hemiplegia and sensory dysfunction were lower in the stable group than in the progressive group (32.73% vs. 55.96 and 3.64% vs. 11.24%, *p* = 0.002 and *p* = 0.047, retrospectively). In addition, the stable group showed a lower proportion of cerebral infarction than the progressive group (42.73% vs. 47.19%); however, the difference was not significant (*p* = 0.529). The proportion of cerebral hemorrhage was significantly lower in the stable group than in the progressive group (45.45% vs. 61.80%, *p* = 0.022).

Before anticoagulation, radiological examinations showed that the stable group had significantly lower proportions of superior sagittal sinus and torcular herophili involvements than those in the progressive group (50.00% vs. 75.28 and 7.27% vs. 17.98%, *p* < 0.001 and *p* = 0.025, retrospectively). Furthermore, the stable group exhibited a lower proportion of inferior sagittal sinus involvement relative to the progressive group (7.27% vs. 8.99%), but the difference was not significant (*p* = 0.659). Patients in the stable group exhibited significantly lower proportions of deep vein and tentorial sinus involvement than those in the progressive group (4.55% vs. 24.72 and 14.55% vs. 34.83%, *p* < 0.001 and *p* = 0.001, respectively). Subsequent analyses revealed that the stable group tended to have lower proportions of transverse sinus, sigmoid sinus, and cortical vein involvements compared to the progressive group. On average, the mean number of occluded sinuses in the stable group was significantly lower than that of the progressive group (2.14 vs. 3.17, *p* < 0.001).

Results shown in Table 1 indicate that pregnancy and puerperium (OR = 5.525, *p* = 0.001), oral contraceptive use (OR = 28.720, *p* = 0.003), coma at admission (OR = 3.890, *p* < 0.001), cerebral hemorrhage (OR = 2.151, *p* = 0.038), and the number of occluded sinuses (OR = 1.708, *p* < 0.001) were independent risk factors predicting progressive SCVST and endovascular or surgical treatment may be considered when clinically necessary.

**Figure 1:**
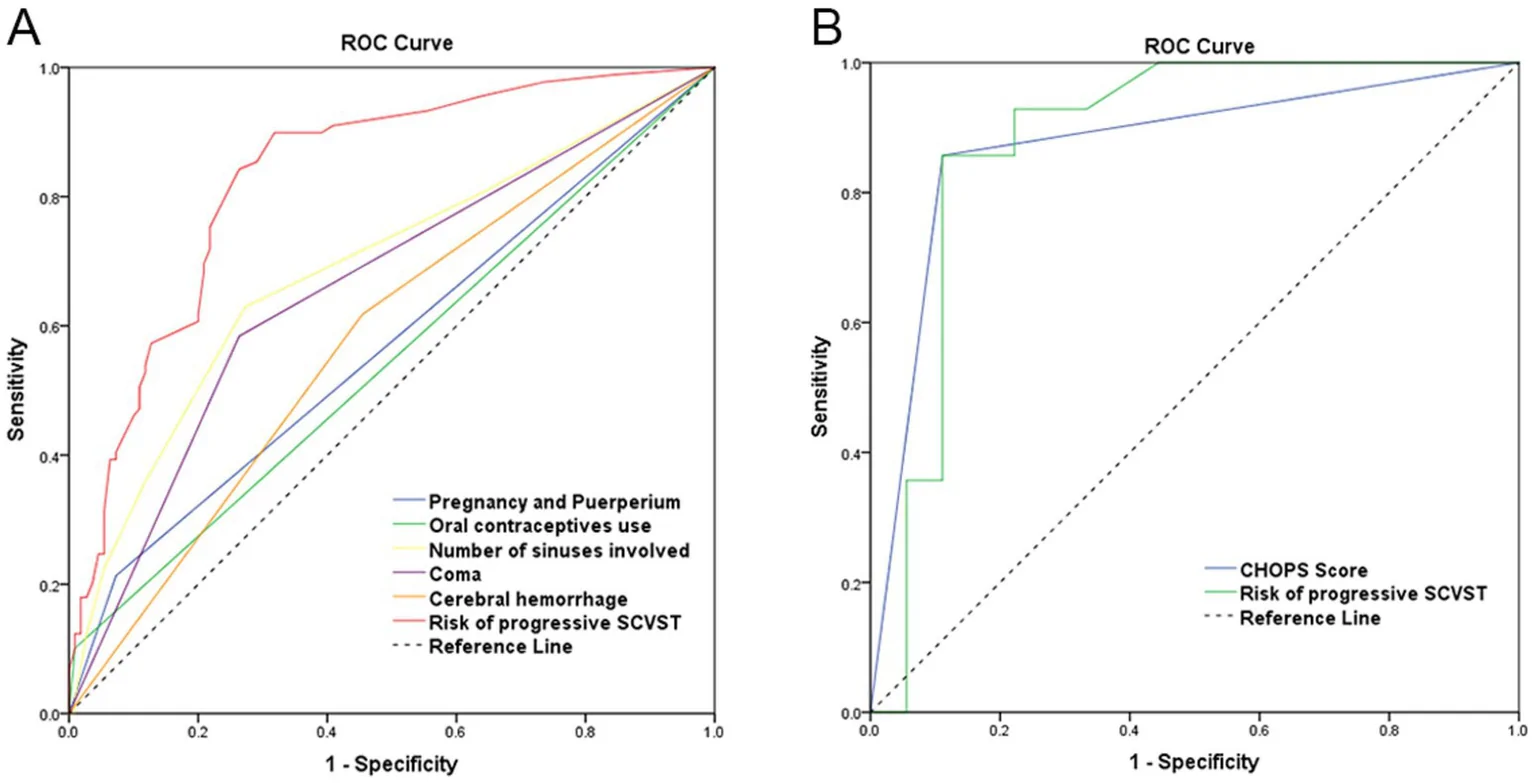
Results of the ROC analysis. **(A)** The original risk score and the five independent factors in 199 SCVST patients. **(B)** The original risk score and the CHOPS score in 32 SCVST patients.

Using the five independent factors identified in the multivariate analysis, we first constructed an original risk score for progressive SCVST based on their corresponding odds ratios (ORs). In this model, the number of occluded sinuses was assigned 1 point, and the remaining factors were weighted according to the relative magnitudes of their ORs. Thus, pregnancy and puerperium were weighted 3.235 points, oral contraceptive use was weighted 16.815 points, coma at admission was weighted 2.278 points, and cerebral hemorrhage was weighted 1.259 points. The ROC curve was used to validate the accuracy of the risk score and the 5 independent factors using data from 199 SCVST patients (Figure 1A). Notably, the AUC value of the risk score was 0.831, which exceeded that of pregnancy and puerperium (0.570), oral contraceptive use (0.546), coma at admission (0.660), cerebral hemorrhage (0.582), and the number of occluded sinuses (0.690). Moreover, the cut-off point of the risk score was 4.247.

To simplify the calculation procedure, we assigned 1 point to each occluded sinuses and used the relative magnitudes of the ORs. Thus, the evaluation system (coma at admission (C, 2 points), cerebral hemorrhage (H, 1 point), oral contraceptive use (O, 17 points), pregnancy and puerperium (P, 3 points), and number of occluded sinuses (S, 1 point per sinus), (CHOPS score)) was developed to simplify the risk calculation (Table 2). We then used an ROC curve to compare the accuracy of the original risk score and our CHOPS score in the database with 32 SCVST patients (Figure 1B). In the 32 SCVST patients, 14 (43.8%) had progression. The AUC of the CHOPS score was 0.873, which was slightly lower than that of the original risk score (0.881). Of the 32 patients, 14 (43.8%) were predicted to be at higher risk. Its 95% confidence intervals were 0.736–1.000, and its sensitivity and specificity at the cutoff point were 0.857 and 0.889.

**Table 2:** Calculation procedure of CHOPS score.

Points	Characteristics
	Oral contraceptive use
0	No
17	Yes
	Pregnancy and puerperium
0	No
3	Yes
1 (per sinus)	Number of sinuses involved
	Coma
0	No
2	Yes
	Cerebral hemorrhage
0	No
1	Yes
Total points	Progression risk
0–4	Low
≥5	High

## 4 Discussion

For SCVST patients without cerebral herniation, systemic anticoagulation is considered the first-line treatment following admission. For patients with progressive symptoms after anticoagulation, endovascular and surgical treatments may be required (6, 7). Therefore, it is imperative to identify patients with progressive symptoms after anticoagulation and administer endovascular and surgical treatments to prevent poor outcomes. In this study, we identified five independent risk factors and established the CHOPS score, which is expected to improve the implementation of individualized treatment.

### 4.1 Coma and cerebral hemorrhage at admission

Clinical evidence has demonstrated that anticoagulation can improve symptoms in patients with hemorrhagic SCVST (4, 7, 13). This effect may be explained by the prevention of thrombus extension, reduction of venous pressure, and decreased risk of rebleeding (13). Nevertheless, anticoagulation may also pose a risk of hemorrhagic worsening, particularly in patients who subsequently require endovascular or surgical treatment. In our CHOPS score, hemorrhagic SCVST patients with coma were assigned at least 4 points, which suggests a substantially increased risk of progression. Notably, coma contributed more to the score than cerebral hemorrhage (2 points vs. 1 point), and its predictive performance was also higher (AUC 0.660 vs. 0.582). These findings indicate that coma may reflect a more severe disease state and a stronger need for timely invasive intervention.

### 4.2 Oral contraceptive use, pregnancy and puerperium

Oral contraceptive use, pregnancy, and puerperium have been proposed as risk factors of CVST, suggesting a possible association between increased estrogen levels and hypercoagulability (13–16). Similarly, in the multivariate results (oral contraceptive use, pregnancy and puerperium), we found that estrogen level was correlated with progressive symptoms, suggesting more cautious management and therapeutic drug monitoring were required for these patients. Notably, multivariate analysis results showed that the OR of oral contraceptive use was 28.720, exceeding that of pregnancy and puerperium (OR = 5.525), which highlighted a pronounced effect of exogenous estrogen on the coagulation system. However, we did not find that females had a significantly higher risk of progressive symptoms, suggesting that physiological estrogen levels have minimal effects on anticoagulation.

### 4.3 Sinus occlusion

Similar to the findings from a previous study (4), deep vein occlusion was a risk factor of progressive symptoms in the univariate results of the present study, indicating that the venous drainage disorder of the thalamus and brainstem was associated with unfavorable outcomes. Moreover, superior sagittal sinus occlusion was a risk factor in our univariate analysis, consistent with reports by Gazioglu et al. (16) However, in the multivariate results, deep vein and superior sagittal sinus occlusions were not significant risk factors. In the proposed CHOPS score, we used the number of occluded sinuses to represent the extent of thrombosis, which indicated that thrombosis volume was associated with the progression of SCVST.

### 4.4 CHOPS score

Finally, we constructed a score for evaluating the risk of progressive symptoms using the ORs of the five independent risk factors. However, the score was difficult to calculate. Consequently, we utilized approximate values of the OR ratios to formulate the CHOPS score for simplified identification of patients. In the validation dataset, the accuracy of the CHOPS score was slightly lower than that of the original score.

## 5 Limitation

This is a single-center retrospective study, and selection bias may affect our results. Thus, our results may only be accurate within the SCVST patients. To apply in clinical practice, a multicenter prospective trial is needed further.

## 6 Conclusion

This retrospective study analyzed the clinical and radiological data of 199 SCVST patients and demonstrated that pregnancy and puerperium, oral contraceptive use, coma at admission, cerebral hemorrhage, and number of occluded sinuses were independent risk factors for progressive symptoms, and these patients tended to receive endovascular and surgical treatments. Moreover, the CHOPS score system comprising five independent risk factors was established. The advantage of this scoring system is that the five factors are easy to obtain and the scoring system is easy to calculate.

## Data Availability

The raw data supporting the conclusions of this article will be made available by the authors, without undue reservation.
